# Marked gender inequity in the invited speakers at the European College of Veterinary Surgeons annual scientific congress 2012–2022

**DOI:** 10.1371/journal.pone.0329147

**Published:** 2025-09-02

**Authors:** Kathryn Pratschke, Poppy Bristow, Alina Paczesna, Ishita Parakh, Jill R. D. MacKay, Fiona Mackay, Kelly Blacklock

**Affiliations:** 1 Royal (Dick) School of Veterinary Studies, University of Edinburgh, Scotland,; 2 Veterinary Specialists Ireland, Clonmahon, Summerhill, Co. Meath, Ireland,; 3 School of Social and Political Science, University of Edinburgh, Scotland; Gulu University, UGANDA

## Abstract

The objective of this retrospective study was to explore gendered equity for invited speakers at the European College of Veterinary Surgery (ECVS) Annual Scientific Meeting between 2012–2022 when compared to speciality demographics for ECVS membership. Our sample populations included the European College of Veterinary Surgeons (ECVS) Diplomate membership, and all invited speakers at their Annual Scientific Meetings between 2012–2022. Data was extracted from Meeting Programs including year, speaker name, session type, and frequency of invitation. Authors were assigned a binary gender using a web-based algorithm to determine gender by a first name. The number and gender of new Diplomates each year between 1993–2023 was obtained from the ECVS Office and used as a comparison group to assess proportional representation amongst invited speakers. We found that women comprised 27% (249/924) of ECVS Diplomates in 2012 and 33.82% (312/924) in 2022. In this decade, there were 913 invited lectures delivered at ECVS Annual Scientific Meetings, 21% (188/913) were delivered by women. Women were particularly under-represented for higher prestige lectures including State of the Art (0%), Pre-Congress wet labs (0%) and Pre-Congress expert-led sessions (15.8%, 15/95). In conclusion, the proportion of invited speakers that were women at ECVS Scientific Meetings between 2012–2022 was 21%, despite women comprising >25% of ECVS Diplomate membership since 2012. Higher prestige sessions were heavily biased towards speakers being men. We suggest proactive commitment is needed to achieve gender equity in speaker invitations across all session types at ECVS Annual Scientific Meetings.

## Introduction

In the past 10–15 years in human healthcare there has been a steadily growing call to address systemic gender bias that impedes equity for women and impacts their career progression [1–9]. The veterinary profession was historically male dominated from the early 1960s to the late 1980s, at which stage equivalent numbers of women were seen at undergraduate level [10]. From this point on, women have grown to dominate in numbers in the profession, comprising over 70% of graduates in the UK and US by the mid 2010s [11]. Between 2010 and 2019, approximately 75% of veterinarians qualifying in the UK were women [12], and in 2021 approximately 80% of the new graduating veterinarians in the USA were women [13]. Despite this changing demographic in veterinary medicine, women remain under-represented in senior management and leadership positions; at higher levels in academia; they continue to receive lower salaries than their male counterparts; career progression in academia is typically slower than for men; and they receive fewer professional awards [8,14–18].

This bears similarities to the ‘leaky pipeline’ phenomenon where women are under-represented at higher levels within Science Technology Engineering Mathematics and Medicine (STEMM) subjects despite being equally or overly represented at the recruitment stage [19]. ‘Leaky pipeline’ phrasing can be critiqued however as implying a passive loss of talent, rather than recognizing structural issues and barriers that affect career progression [8,13,17,19]. The European Universities Association definitions of diversity, inclusivity and equality consider diversity as the demographic and social composition of a group, encompassing factors such as sex, gender, age, sexual orientation, ethnicity and cultural associations, religions, health conditions and socio-economic background [20]. Inclusivity refers to the actions taken to ensure a diverse population feels valued. Equity is then the end goal for equality agendas, as it acknowledges that individuals have different starting points, and that specific barriers are faced by some which need to be overcome for meaningful engagement. Recognising that there may be unique barriers to women in veterinary surgery is therefore an important step on the road to equity.

Invitations to speak at conferences help enhance an individual’s reputation and provide opportunities for collaboration and networking. They also allow role-modelling and mentorship opportunities. Research across a range of specialty conferences in human healthcare shows widespread under-representation of women amongst the ranks of invited speakers [3,4,7,9,21–38]. Women are also less likely to deliver higher prestige or keynote lectures, more likely to deliver shorter lectures, and more likely to be asked to speak on non-technical subjects e.g. work-life balance, resident training, medical education and medical ethics [7,9,22–24,29,34,36,38].

Cultivating gender equity as part of an overall diversity strategy helps foster innovation, challenge assumptions, and promote problem solving [39]. The term gender is typically taken to mean the social construct of male, female and gender-diverse people [40]. The World Health Organisation defines gender as “the characteristics of women, men, girls and boys that are socially constructed. This includes norms, behaviours and roles associated with being a woman, man, girl or boy, as well as relationships with each other. As a social construct, gender varies from society to society and can change over time”. Gender has implications throughout life, often intersecting with other markers of inequality such as ethnicity, socioeconomic status, age, and sexual orientation.

A study evaluating speaker introductions at the American College of Veterinary Surgeons (ACVS) Scientific Meeting in 2018 found a trend for presenters that were women to be introduced informally and without professional titles compared to their male counterparts although the difference did not reach statistical significance [41]. To date however, there are no publications evaluating invitations to lecture for veterinary specialty conferences. Our objective was to explore gendered equity for invited speakers at ECVS Annual Scientific Meetings between 2012–2022 when compared to demographics for ECVS Diplomate membership. Our hypothesis, based on available literature in comparable fields, was that women would be proportionately under-represented as invited speakers, particularly in higher prestige sessions.

## Materials and methods

The Human Ethics Review Committee (Ref: HERC 23_056) for the Royal (Dick) School of Veterinary Studies, University of Edinburgh approved this project. Ethical approval was granted for research investigating female representation and gendered inequity at European College of Veterinary Surgery Annual Scientific Meetings (2012–2022), with the Committee providing a favourable ethical opinion of the above research. The Ethics Review Committee waived a need for the study to gain specific consent from individuals listed as Conference speakers on Proceedings on the basis that we adhered to the ethics guidelines of the Association of Internet Researchers when mining for additional data from people on the list (to assign gender), and we used only data which was on publicly available veterinary based websites. There were two main sources of data for our analysis. The first was the European College of Veterinary Surgeons (ECVS) Diplomates list as of May 25th, 2023, provided by the ECVS Secretarial Office in anonymized format. This list contained Diplomate gender, year of registration, specialty (small or large animal), and surgical emphasis (orthopedic, soft tissue etc). Invited speakers were bench-marked against the proportion of female Diplomates in the ECVS membership, measured from 1993–2023. This is consistent with methodology used in previous publications, and we felt it was the most appropriate way to assess proportional representation [3,7,9,28–31,33,35,37,38,42]

The second source was Conference Scientific Programs from 2012−2022 (note, ECVS Conference was not held in 2020 due to the Covid-19 pandemic). Data recorded from Scientific Programs included first and last name of invited speakers, specialty, surgical emphasis, title and type of presentation. If a first name was not available, and the individual was not listed in the European Board of Veterinary Specialisation (EBVS) or other Specialty College database, the name was further searched in Google to identify individuals via information on institutional websites, LinkedIn profiles, journal publications, and other websites.

Datasets required data cleaning with an element of subjectivity. For the Diplomates list, specialty was identified as Small Animal or Large Animal. Surgical Emphasis was reduced to three categories – none specified/general, orthopedic, or soft tissue.

For ECVS Conference datasets, one author (KP) determined whether a presentation was from an invited speaker or accepted through blinded review. This gave a subset of the ECVS Conference data with invited presentations between 2012–2022, further categorized by gender and session type. Finally, we looked at career length of invited speakers by categorizing each speaker based on the period of time since they passed the ECVS Board-certifying examination to become an ECVS Diplomate (or the ACVS Board-certifying examination to become an ACVS Diplomate) and their invitation to speak at ECVS. Diplomate status was defined as indicating an individual that had passed their Board-certifying examinations successfully and become an ECVS Diplomate.

- Early career – achieved Diplomate status 5 years or less previous to invitation
- Experienced – achieved Diplomate status more than 5 years previous to invitation
- Other equivalent expert – Comparable level of expertise to an Experienced Diplomate e.g. Diplomates from other specialty colleges with 5 or more years post Diploma experience previous to invitation, or individuals holding full Professorships.

### Gendering diplomates

Following methodology described in several previous studies [43–48], we used a web-based algorithm to determine gender by a first name (https://gender-api.com). Gender-API has been evaluated in comparison to other gender detection tools including NamSor (https://namsor.app/), genderize.io (https://genderize.io/), Wiki-Gendersort (https://github.com/nicolasberube/Wiki-Gendersort), and is consistently described as one of the most accurate available tools [49–51]. Probabilistic name-to-gender inference services have been widely used in marketing and political research and more recently in social and medical science. Online services such as this rely on large, regularly maintained databases of names which are drawn from publicly available sources such as government records. The Gender-API database contains approximately 6.1 million names from 191 countries. It evaluates a name and assigns a binary gender category (male or female) with an accuracy rating from 0–100 per the Gender-API’s probability rating. Accuracy is calculated by the number of records in the training database, e.g. if there are 100 samples of a given name and 96 are recorded female, the accuracy for that name will be 96%, with an accuracy score of 96. A threshold for low accuracy was defined as <70 [43] at which point the name was manually reviewed to assign gender, based in a professional profile. If gender could not be assigned, the name was excluded from further analysis.

### Statistical preface

This paper utilises a branch of statistics that may be unfamiliar to the reader, this section serves as a primer to enable readers to evaluate the results. Most readers are familiar with Frequentist statistics where p-values are used to denote a finding of interest. For example, a generalised linear model might look to explain how much variation in a response (y) can be accounted for by an explanatory variable (x). As a note, this is always what statistics tries to do, assign the variation observed in y to various explanatory variables, and this is very suited to the linear model framework, hence why some statisticians will use a linear model in place of most traditional statistics [52].

As we had a dataset of many hundreds of individuals, we were conscious of the ability to find significant results through numbers alone [53]. ‘Significance’ is a purview of Frequentist statistics, where probability is equivalent to frequency, and the conclusions are extrapolated from an event’s frequency within the sample data. Frequentist statistics use p-values to denote whether a finding is interesting, although p-values have recently been the subject of critique for being an overly simplistic application of the Frequentist approach [54]. While examination of effect size can (and should) go some way to mitigate this, there are other limitations of the Frequentist approach, such as assumptions regarding independence of samples, which may not be fully recognised in our dataset. For example, as more women entered the profession, the profession may become more attractive to women. Additionally, while Frequentist statistics allows you to reject a null hypothesis with a significant p-value, a non-significant p-value does not allow you to accept the null hypothesis.

Recent advances in computing power make it far easier to additionally calculate Bayesian inferences for a given model [55]. Bayesian approaches are more robust to assumptions of data and generally can be considered as putting a probability on the difference between two groups being non-zero [56]. A linear model calculated through Frequentist or Bayesian inference will still provide estimates of the coefficient for each explanatory term in the model. For a numerical explanatory variable, the estimate of the coefficient can be thought of as ‘what does one unit increase in this variable do to the response variable?’ For categorical variables (such as specialist status), the estimate of the coefficient describes what a change in the status of that variable does to the response, e.g. what does being a specialist versus not being a specialist do to the likelihood a speaker will be a man or a woman? Frequentist models generate estimates of the coefficient alongside a standard error and associated with these estimates we have p-values, which are intended to indicate whether a term may be of interest. Bayesian models generate a posterior distribution of the coefficient, essentially a range of the most likely estimates at a 95% Credible Interval. We follow the Sequential Effect eXistence and sIgnificance Testing (SEXIT) framework [57] and report the median of the posterior distribution and its 95% CI, the probability of the effects direction, the probability of significance (e.g. if the effect is over a threshold), and the probability of it being large (e.g. if the effect is over a threshold). Note that probability of significance and probability of size are both based on whether the probability of direction reaches a threshold. Convergence and stability of Bayesian sampling is assessed via R-hat (< 1.01 [58]) and the Effective Sample Size (ESS > 1000 [59]). The Bayesian framework gives us a more detailed understanding of whether our data fits with a large effect or not. Other than not reporting a p-value, the interpretation of the model is similar to the Frequentist framework in that you have a coefficient, a range around the coefficient, and instead of a single estimate of whether the effect is interesting (a p-value) you have a series of probabilities of the effect being interesting and the size of the effect.

### Statistical analysis

To better understand what factors impacted the gender of a given Diplomate or invited speaker, we used a logistic regression in a Bayesian framework to estimate the probability of a speaker being a woman, under a given set of conditions. Models were built in both Frequentist and Bayesian frameworks, but for brevity we report only the Bayesian model as we believe it is the more relevant of the two for this dataset. To select the final model, we compared the Bayes R2 as a measure of the model’s explanatory power and examined the probability of significance of individual terms. Gender of the speaker was the response variable, with man coded as the default and woman as the change of interest.

For variation in invited speaker gender, explanatory variables were Diplomate Status (True/False), career stage, specialty, session type, and conference year (with 2012 coded as Year 1). All analyses were performed in R (R Core Team 2023, version 4.2.3 (“Shortstop Beagle”)), and made use of a variety of available packages [59–62]. Analysis Code is Available here: https://osf.io/rpb3z/?view_only=37545e161aa640f483c60d079e2fa0eb. Further information is available in the Supplementary files (S1 Table and S2 Table).

## Results

### ECVS membership

**Fig 1:**
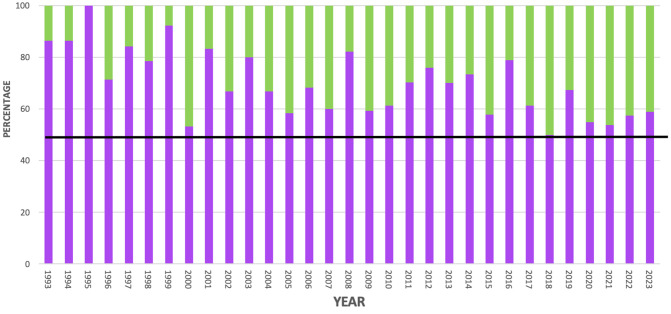
Proportion of 924 ECVS Diplomates who were women each year between 1993–2023. Green represents female, purple represents men, the black line represents 50%.

Across 30 years (1993–2023), 33.8% (312/924) of Diplomates in total were women. The number of new Diplomates that were women varied by year, from none in 1995 to a maximum of 50% (22/44 new Diplomates) in 2018 (Fig 1). Distributions were skewed across surgical emphases; only 20% 53/271) of Diplomates with an orthopedic emphasis were women compared to 39% (205/519) with general and 40% (54/134) with soft tissue (Table 1).

**Table 1:** Proportion of the 924 Diplomate registrations with ECVS between 1993 and 2023, grouped by Diplomate details and gender.

Trait	Description	Gender	Number	% women from overall
Diplomates (n = 924)					
OVERALL	**Diplomate**	**Women**	**312**	**34%**
	Diplomate	Men	612	66%
SURGICAL EMPHASIS	**None/General**	**women**	**205**	**39%**
	None/General	men	314	61%
	**Orthopedic**	**Women**	**53**	**20%**
	Orthopedic	Men	218	80%
	**Soft Tissue**	**Women**	**54**	**40%**
	Soft Tissue	Men	80	60%
SPECIALTY	**Large animal surgery**	**Women**	**27**	**43%**
	Large animal surgery	Men	36	57%
	**Large animal surgery (equine)**	**Women**	**83**	**33%**
	Large animal surgery (equine)	Men	170	67%
	**Small animal surgery**	**Women**	**202**	**33%**
	Small animal surgery	Men	406	67%

At the intercept (corresponding to the year 1993 and a having a general surgical emphasis), the model predicted that 21% (95% CI 14%, 30%) of Diplomates would be women. From 2002−2022, each year increased the odds probability of a Diplomate being a woman by 4% (95% CI 2%, 6%, prob_significance_ 0%). Having an orthopedic emphasis reduced the odds of a Diplomate being a woman by 48% (95% CI, 65%, 23%, prob_significance_ > 99.9%), whereas a soft tissue emphasis increased the odds by 43% (95% CI −7%, 118%, prob_significance_ 89.3%).

### Gender split in ECVS invited speakers

**Fig 2:**
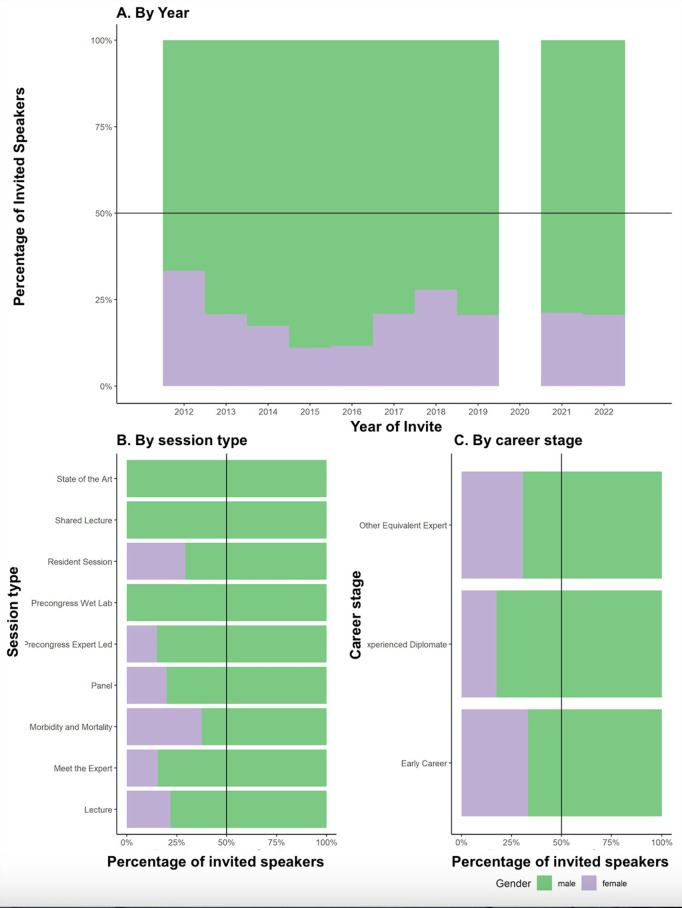
Proportion of 913 ECVS Invited speaker sessions which were delivered by women by A) year, B) Session Type and C) Career Stage. The black line represents 50%. Green indicates women invited to deliver the session; purple indicates men.

Percentage of women speakers by year are illustrated in Fig 2. There were 913 sessions delivered by invited speakers from 2012–2022, of which 21% (188/913) were delivered women (Fig 2A). The 913 sessions were delivered by 415 unique individuals; we were unable to reliably characterize gender for 11/415 (2.6%) of all invited speakers. The percentage of invited speakers that were women in 2012 was the highest across the decade, at 37.7% (26/69). Over the subsequent years up to 2022, the percentage invited speakers that were women was 23% (17/74), 18.5% (15/81), 11.3% (8/71), 12.2% (10/82), 18.3% (24/131), 29% (22/74), 21.9% (21/96), 21.1% (19/90) and 19% (18/94) respectively. The most common session type was a lecture (n = 710; 22% (156/710) of which were presented by women) followed by Pre-Congress Expert Led (n = 106; 15% (16/106) of which were presented by women), Meet the Expert (n = 45; 16% (7/45) of which were presented by women), Resident Education Sessions (n = 17; 29% (5/17) of which were presented by women), Panels (n = 10; 20% (2/10) of which were presented by women), State of the Art (n = 9; none were presented by women), Morbidity and Mortality Rounds (n = 8; 37% (3/8) of which were presented by women), Shared Lectures (n = 4; none were presented by women), and Pre-Congress Wet Labs (n = 4; none were presented by women). Men outnumbered women as invited speakers in every session type (Fig 2B). Sixteen invited speakers were at Early Career Stage (31% (5/16) of which were women), 670 were Experienced Diplomates (17% (119/670) of which were women), and 15 were Other Equivalent Expert (31% (5/15) of which were women) (Fig 2C). A summary of the gender split in invited speakers across Diplomate status, specialty, session type, and surgical emphasis, is in Table 2.

**Table 2:** Proportion of women invited to speak at ECVS from 2012–2022 across Session Type, Large/Small Animal, and surgical emphasis.

TRAIT	GENDER	N^1^	%
**Session Type**			
• Lecture	Men	555	78%
	**Women**	**155**	**22%**
• Meet the Expert	Men	38	84%
	**Women**	**7**	**16%**
• Morbidity and Mortality	Men	5	62%
	**Women**	**3**	**38%**
• Panel	Men	8	80%
	**Women**	**2**	**20%**
• Pre-Congress Expert Led	Men	90	85%
	**Women**	**16**	**15%**
• Pre-Congress Wet Lab	Men	4	100%
	**Women**	**0**	**0%**
• Resident education session	Men	12	71%
	**Women**	**5**	**29%**
• Shared Lecture	Men	4	100%
	**Women**	**0**	**0%**
• State of the Art	Men	9	100%
	**Women**	**0**	**0%**
**Large or Small Animal**			
• Relevant to both Large and Small2	Male	16	84%
	**Women**	**3**	**16%**
• Large Animal only	Men	255	77%
	**Women**	**78**	**23%**
• Small Animal only	Men	452	81%
	**Women**	**106**	**19%**
• Other3	Men	2	67%
	**Women**	**1**	**33%**
**Surgical emphasis**			
• General	Men	53	74%
	**Women**	**19**	**26%**
• Orthopedic	Men	384	82.4%
	**Women**	**82**	**17.6%**
• Soft Tissue	Men	284	77%
	**Women**	**85**	**23%**
• Other^2^	Men	4	67%
	**Women**	**2**	**33%**

^1^N – number.

^2^Topics such as surgical hand preparation, or antimicrobial resistance, that were not species-specific were in this classification.

^3^The designation ‘other’ was used if information was not recorded that allowed allocation to a specific group.

### Repeated invitations

**Fig 3:**
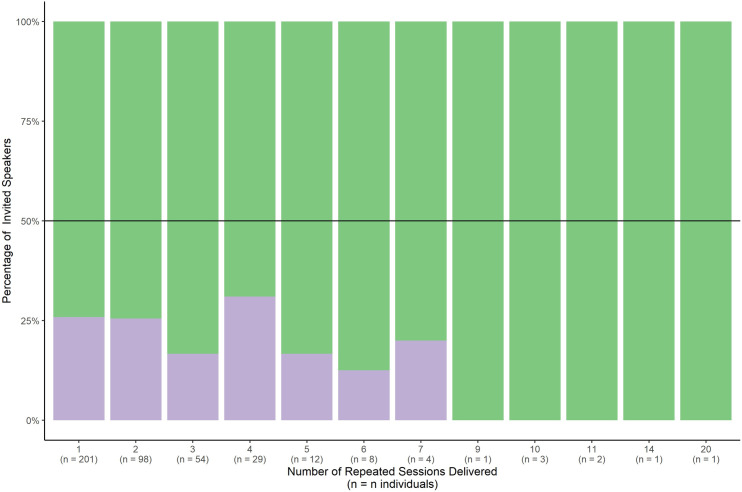
This figure shows the proportion of Invited Speakers who were invited multiple times. This ranged from 201 individuals who were invited to speak once, through to one man who was invited to speak 20 times. Green indicates women invited to speak; purple indicates men. The black line represents 50%.

There were 913 sessions delivered by invited speakers from 2012–2022, with 415 unique individuals delivering these sessions. Men delivered on average 1.02 lectures per individual, and women presented 1.97. Of these 415 invited presenters, 201 were invited once (of which 26% (52/201) were women), 98 were invited twice (of which 24.5% (24/98) were women), 54 were invited three times (of which 16.6% (9/54) were women), and 29 were invited four times (of which 31% (9/29) were women). Eight men accounted for 9% (82/913) of all invited sessions presented and 49 men accounted for 28% (255/913) of all invited sessions presented. Of note, this figure surpasses the 21% (191/913) of all invited sessions delivered by women (Fig 3).

### Predicting the likelihood an invited speaker will be a man or a woman

The best model to predict the likely gender of an invited speaker, meaning the probability that an invited speaker would be a man/woman, included year, Diplomate status, career stage, and surgical emphasis. The model’s explanatory power was weak however (R^2^ = 0.04), meaning that year, Diplomate status, surgical emphasis and career stage do not adequately explain or influence likely speaker gender and other factors must be involved. Looking at an Early Career ECVS Diplomate in 2012 with a general surgical emphasis, the predicted probability an invited speaker would be a woman was 44% (95% CI 19%, 71%) and each year increased the probability by < 1% (95% CI −5%, 6%, prob_significance_ = 0.05%). Being an Experienced Diplomate reduced the probability an invited speaker would be a woman by 71% (95% CI −90%, −14%, prob_significance_ 98.15%). Having an orthopedic surgical emphasis was associated with a 17% reduction in probability the speaker would be a woman (95% CI, −55%, 54%, prob_significance_ = 62.98%) but having a soft tissue emphasis gave a 4% increase (95% CI, −44%, 93%, prob_significance_ = 44.02%).

## Discussion

Our study found women were consistently under-represented as invited speakers at ECVS Annual Scientific Meetings from 2012–2022 when compared to specialty demographics. The disparity was particularly marked in more prestigious sessions, reflecting previous studies from medical specialty conferences in human healthcare [3,4,7,9,21–25,27–34,36–38,42,63,64]. Therefore, we accept our hypothesis that women were proportionately under-represented as invited speakers at ECVS Annual Scientific Meetings from 2012–2022, particularly for higher prestige sessions.

From 2012–2022, women made up 27–33.2% of ECVS Diplomate membership but accounted for delivery of only 21% of invited lectures at the Annual Scientific Meetings in the same period. If women were proportionately represented as invited speakers these meetings, then allowing for time lag bias we should see a steadily increasing percentage of women presenting invited sessions from 2012–2022, approaching 33% by 2022 [28]. Time lag bias recognises the fact that a relatively smaller proportion of women were completing ECVS training programs and becoming ECVS Diplomates in the early years of the College. We expect to see a trend towards this imbalance ‘self-correcting’ over time as the number of women achieving ECVS Diplomate status increases and they develop their careers i.e. there would be a natural time lag before reaching proportional representation.

When we consider high prestige sessions, no State-of-the-Art lecture was delivered by a woman; women comprised 15% of invited speakers for Pre-Congress expert-led workshops, and 0% for Pre-Congress Wet Labs. A higher percentage of women who presented lectures delivered multiple lectures compared to men. As mentioned in the results section, men delivered on average 1.02 lectures per individual, and women presented 1.97. As a result, fewer women benefitted overall from invitations to speak. As a theoretical example, if 10 lectures were delivered by men, then working on 1.02 lectures/speaker on average, this means 9.8 men have benefited from an invitation to speak but working on 1.97 lectures/speaker on average for women, it means that 5.1 women have benefitted from invitations. This may suggest a lack of variety in the women approached to lecture at ECVS meetings, and perhaps that there is a perception there is a smaller pool of appropriately qualified women when compared to men. During the period 2012–2022 there were no formal guidelines in place for ECVS Program Organising Committees regarding the selection process for invited speakers, beyond it being their duty to develop the program for Annual Scientific Meetings for Final Approval by the Board of Regents. A call for speakers to self-nominate was put out by the Programme Committee in 2024; it remains to be seen what the gender divide will be amongst those who self-nominate, but the available evidence suggests women are typically more reticent about putting themselves forward [65,66]. A pro-active commitment to diversity and inclusion of invited presenters made by the Board of Regents as well as the Programme Committee could be useful to encourage inclusivity.

Under-representation of women as invited speakers can reinforce unconscious or implicit bias that fewer appropriately qualified or expert women are available to speak when compared to men in the same specialty. This concept links directly to the oft-used argument that lower numbers of invited women as speakers simply reflects historical differences in the numbers of men and women as trainees and is a product of time lag bias. However, for this argument to be valid we should see a consistent difference in career length between the men and women invited to speak. If women were under-represented due to a relative paucity in 2012, then based on ECVS membership demographics we should see a steady increase from 2012–2022 as more women became board-certified and developed their careers. We would also expect invited speakers that are men to consistently have longer, more established careers than invited speakers that were women. On the contrary, our data shows that numerically more early career men were invited to speak than women. Time lag bias should result in numerically more early career women receiving invitations. The ratio of men:women in the Experienced Diplomate speakers from 2015 on remained between 4.5:1 and 4.2:1; again, if time lag bias was significantly impacting speaker invitations, we should see this ratio shifting over time. Interestingly, low rates of woman as invited speakers irrespective of career length is in keeping with the findings of a similar 10-year review of Plastic Surgery Conferences published in 2022 [28]. Our study showed the modelled impact of each additional year from 2012 onward was very small, suggesting that even discounting the possibility of systemic biases, simply waiting for the situation to resolve itself with time will take decades. As such, there is an argument for a pro-active approach to be adopted.

Academic meetings serve an important role for veterinary specialties by disseminating new findings, opinions, and techniques; they allow communication of current trends; and are perceived as identifying those seen as leaders within their field. If women are consistently under-represented, particularly for more prestigious sessions, the picture conference delegates see every year is biased towards seeing more men occupy influential positions. This may feed into unconscious or implicit bias, reinforcing a perception that men are better surgeons, more knowledgeable experts and natural leaders in our field. The presence of this type of unconscious or implicit bias has previously been demonstrated in studies in the human medical literature [67,68].

Research shows that even where faculty and leadership figures in medicine have similar scholarly activity, there is still a clear gender discrepancy in career progression [6,8,17]. A recent North American study found that in veterinary medicine men were 2.5 times more likely to be in Associate/Full Professor positions than women, while women were concentrated at lower academic levels, received lower salaries, and took longer to be promoted than men [8]. A global study noted that in Australia and New Zealand, 75% of Professorships were held by men, in the USA and Canada this figure was 80% and in Europe, veterinarians at senior levels in academia were twice as likely to be men than women [17]. It is important that women have equal access to activities and opportunities, such as invitations to lecture at international conferences, that can help further their careers and facilitate their prospects for promotion.

A relative lack of women as invited speakers at international conferences also impacts the nature of the role models presented to younger surgeons and aspiring surgeons. The less diverse the spectrum of strong, intelligent, and impressive role models available, the less diverse the pool of those aspiring to become specialists is likely to be. Diverse groups are better able to identify and cope with problems, they assimilate new information more readily, and they perform at a higher level when faced with adversity [9,39]. Strong women as role models are particularly important for progression and retention of women within a specialty [69]. In the specialty of veterinary surgery, in 2019 women comprised 37% of all American College of Veterinary Surgeons (ACVS) Diplomates, but only 3/52 (5.8%) ACVS Presidents were women and only 2/49 (4.1%) ACVS Achievement Awards were given to women [8]. In 2023, women comprised 33.8% of ECVS Diplomates, and 5/30 (16.7%) ECVS Presidents were women. According to information on the ECVS website (www.ecvs.org), the Inaugural Special Achievement Award in 1995 was presented to a woman, the ECVS Secretary at the time, and two subsequent awards were given to a charitable foundation, and to the ACVS, rather than to an individual. To date, two Distinguished Service awards have been awarded, both to men.

The first necessary step for any change is to characterize the current situation and identify factors that contribute to lack of equity. Several studies have suggested that when women are involved in conference organizing committees, or occupy leadership positions within a specialty college, the gender balance of speakers improves. although this is not a universal finding [4,7,22,24,28,32,37]. Conference committees, regardless of make-up, should be alert to the risk of convenient habitual invitations and instead make active efforts to identify all suitably qualified and expert speakers. Despite suggestions that women are more likely to decline speaker invitations, that they are less well qualified, and less interested in taking up speaking engagements due to an ambition gap between the genders, none of these ideas are supported by evidence and more likely reflect unconscious bias [5,17,21,26,70,71].

Those currently in senior and leadership positions within specialty Colleges such as ECVS could also engage in critical introspection regarding the degree to which their behavior may contribute, albeit unwittingly, to perpetuating gender bias. Importantly, responsibility does not lie solely with men, but also with women in senior positions who exhibit unconscious bias [72–75]. The ability to form collegial relationships and become part of a beneficial professional network should be available to all.

Implicit in the argument for gender equity in speaker invitations is that we do not seek to actively under-represent men as speakers, nor to suggest they should not be invited to speak on their own merits. Diversity of inclusion does not mean compromising on excellence [71]. Rather, we suggest that equally qualified and experienced women, should be equally likely to receive an invitation to lecture, and our data suggests this does not currently happen.

With this study, we aimed to establish foundational information and provide a basis for future research. The retrospective nature of our study meant we could not assess the impact of women on conference organizing committees as this information was not readily available. We only addressed gender, other demographic indicators of diversity including race, socioeconomic status, religion and sexual orientation were not included. These intersectionalities are very important, however it was beyond the remit of the current study to interrogate this level of granular detail. We considered only binary gender archetypes of male and female, which does not allow for non-binary, non-conforming or transgender individuals. We elected to focus on binary gender for this study based on the general lack of research regarding women in veterinary conferences, but also because it is consistent with contemporaneous veterinary publications on the topic, making comparison more appropriate. There were also practical restraints in terms of time, availability and accessibility of data, and allocation of resources. Accrual of more granular self-reported demographic data is not currently standard within veterinary specialty colleges, and this would be required to further assess intersectionality.

We used the online name-to-gender tool Gender-API to allocate binary gender to our dataset of speakers, with a 70% cutoff for accuracy as previously reported [43,44,46,48]. Strengths of Gender-API include its accessibility, ability to assign gender with a high accuracy for most names, and ability to use it on large datasets with high processing speed [49,50]. There are also weaknesses, including assignation of gender on a binary scale, difficulty assigning gender to some names (often non-English), and accuracy below 70% for some names. This necessitated manual checks to confirm gender with higher certainty and identify those where gender was misassigned. In total, we were unable to reliably categorize gender for 2.6% (11/415) of all invited speakers between 2012–2022, which we believe is an acceptably low level to not impact our results significantly.

We believe our findings provide a meaningful, evidence-based step to characterizing the nature and extent of gender bias in the specialty of veterinary surgery. The ECVS Annual Scientific Meetings between 2012–2022 have consistently under-represented the voice of women in our specialty, from an empirical perspective as well as in the context of proportional representation compared to ECVS Diplomate membership. We hope the information we provide with this study will encourage an active commitment to diversity of inclusion in speaker invitations and topic assignment for future conferences. Currently ECVS procedures state that the “…task of the PC (Programme Committee) is to develop a programme for each ASM (Annual Scientific Meeting)…If pre-congress courses are to be held, they should also be coordinated by the Chair in close collaboration with the Local Organising Committee”. There is no rubric or standard protocol in place to help guide speaker selection, so it is possible, if not probable, that invitations are influenced by who is known to the committee, through visibility from previous speaking engagements, being part of a specific professional network, or having publications with high visibility. Introduction of a rubric or standard protocol in addition to making an active commitment to diversity and equality of opportunity could be a useful step towards improving equity.

## Supporting information

S1 Table Model parameters for the logistic model predicting Diplomate Gender (Female = 1) using a Bayesian framework with year and surgical emphasis. Model estimated using MCM sampling with 4 chains of 2000 iterations and a warmup of 1000 iterations.
(DOCX)

S2 Table Model parameters for the logistic model predicting Invited Speaker Gender (Female = 1) using a Bayesian framework with Year, Diplomate Status, Specialism, and Career Stage. Model estimated using MCM sampling with 4 chains of 2000 iterations and a warmup of 1000 iterations.
(DOCX)
