# Supplementary material for: Marked gender inequity in the invited speakers at the European College of Veterinary Surgeons annual scientific congress 2012–2022
Source: PLoS One. 2025 Sep 2;20(9):e0329147. doi: 10.1371/journal.pone.0329147 (PMC12404438; doi:10.1371/journal.pone.0329147)
Supplement: S1 Table — Model estimated using MCM sampling with 4 chains of 2000 iterations and a warmup of 1000 iterations. (DOCX) [file pone.0329147.s001.docx]

Supplementary Table 1: Model parameters for the logistic model predicting Diplomate Gender (Female = 1) using a Bayesian framework with year and surgical emphasis. Model estimated using MCM sampling with 4 chains of 2000 iterations and a warmup of 1000 iterations*.*

| **Parameter** | **Median Effect** | **Odds Ratio** | **95% Lower CI** | **95% Upper CI** | **Probability of**  **Direction** | **R-hat** | **Effective Sample Size** | **Prior Distribution** | **Prior Location** | **Prior Scale** |
| --- | --- | --- | --- | --- | --- | --- | --- | --- | --- | --- |
| Intercept | -1.30 | 29%  (Probability) | -1.81 | -0.83 | 1.00 | 1.00 | 2662.92 | normal | 0 | 2.50 |
| Year | 0.04 | 1.04 | 0.02 | 0.06 | 1.00 | 1.00 | 2946.75 | normal | 0 | 0.27 |
| Surgical Emphasis Orthopedic | -0.65 | 0.52 | -1.05 | -0.26 | >0.99 | 1.00 | 2867.13 | normal | 0 | 5.498 |
| Surgical Emphasis Soft Tissue | 0.36 | 1.43 | -0.06 | 0.79 | 0.95 | >0.99 | 3315.76 | normal | 0 | 7.09 |

CI – confidence interval
