# Supplementary material for: Marked gender inequity in the invited speakers at the European College of Veterinary Surgeons annual scientific congress 2012–2022
Source: PLoS One. 2025 Sep 2;20(9):e0329147. doi: 10.1371/journal.pone.0329147 (PMC12404438; doi:10.1371/journal.pone.0329147)
Supplement: S2 Table — Model estimated using MCM sampling with 4 chains of 2000 iterations and a warmup of 1000 iterations. (DOCX) [file pone.0329147.s002.docx]

Supplementary Table 2: Model parameters for the logistic model predicting Invited Speaker Gender (Female = 1) using a Bayesian framework with Year, Diplomate Status, Specialism, and Career Stage. Model estimated using MCM sampling with 4 chains of 2000 iterations and a warmup of 1000 iterations.

| **Parameter** | **Median Effect** | **Odds Ratio** | **95% Lower CI** | **95% Upper CI** | **Probability of**  **Direction** | **R-hat** | **Effective Sample Size** | **Prior Distribution** | **Prior Location** | **Prior Scale** |
| --- | --- | --- | --- | --- | --- | --- | --- | --- | --- | --- |
| Intercept | -0.26 | 44% (Probability) | -1.40 | 0.83 | 0.68 | 1.00 | 2746.61 | normal | 0 | 2.50 |
| Year | <0.01 | 1 | -0.05 | 0.06 | 0.57 | >0.99 | 3500.59 | normal | 0 | 0.78 |
| Career Stage (Experienced Diplomate) | -1.22 | 0.29 | -2.17 | -0.19 | 0.99 | 1.00 | 2463.55 | normal | 0 | 5.99 |
| Career Stage (Other Equivalent Expert) | -0.11 | 0.9 | -1.09 | 0.94 | 0.59 | 1.00 | 2528.43 | normal | 0 | 6.24 |
| Diplomate Status (False) | -0.79 | 0.45 | -1.42 | -0.19 | 0.99 | 1.00 | 3610.67 | normal | 0 | 7.87 |
| Specialism Neurosurgery | -1.21 | 0.3 | -2.56 | -0.10 | 0.98 | 1.00 | 3119.60 | normal | 0 | 12.47 |
| Specialism Orthopedic | -0.20 | 0.82 | -0.79 | 0.42 | 0.74 | 1.00 | 2620.92 | normal | 0 | 5.00 |
| Specialism  Soft Tissue | 0.05 | 1.05 | -0.53 | 0.65 | 0.56 | 1.00 | 2547.2 | normal | 0 | 5.09 |

CI – confidence interval
